# Generation of Bona Fide Human Induced Trophoblast Stem Cells by Direct Reprogramming of Term Umbilical Cord Cells

**DOI:** 10.3390/ijms26010271

**Published:** 2024-12-31

**Authors:** A. Jantine van Voorden, Souad Boussata, Remco Keijser, Marloes Vermij, Muriel K. Wagner, Wessel Ganzevoort, Gijs B. Afink

**Affiliations:** 1 Reproductive Biology Laboratory, Amsterdam University Medical Center Location University of Amsterdam, Meibergdreef 9, 1105 AZ Amsterdam, The Netherlands; 2 Amsterdam Reproduction and Development Research Institute, 1105 AZ Amsterdam, The Netherlands; 3 Department of Obstetrics and Gynaecology, Amsterdam University Medical Center Location University of Amsterdam, Meibergdreef 9, 1105 AZ Amsterdam, The Netherlands; 4 Amsterdam Institute for Immunology and Infectious Diseases, 1105 AZ Amsterdam, The Netherlands

**Keywords:** direct reprogramming, iPSC, trophoblast stem cell, placenta

## Abstract

Placentation disorders, including severe preeclampsia and fetal growth restriction, have their origins in early pregnancy, whereas symptoms typically present later on. To investigate the pathogenesis of these diseases, there is a need for a reliable in vitro model system of early placenta development with known pregnancy outcomes. Therefore, we optimized the generation of human induced trophoblast stem cells (iTSCs) from term umbilical cord, enabling non-invasive collection of patient-derived material immediately after birth. Using a direct reprogramming approach previously described for dermal fibroblasts, we investigated the effects of three supplements (A-485, BMP4, and EPZ-6438) to assess their potential to enhance iTSC induction. The generated iTSCs fulfilled the criteria for bona fide first-trimester trophoblasts and exhibited key functional capacities, including long-term self-renewal, differentiation into hormone-producing syncytiotrophoblasts and invasive extravillous trophoblasts, and the formation of organoids. Furthermore, transcriptomic analysis revealed high similarity between the generated iTSCs and trophoblast stem cells derived from first-trimester placental tissue. The supplements did not improve the generation of iTSCs. In conclusion, we successfully generated bona fide iTSCs from term umbilical cord using a direct reprogramming approach, providing a robust and clinically relevant model to study early placentation mechanisms in patient-derived trophoblasts.

## 1. Introduction

The introduction of human trophoblast stem cells (TSCs) [1] and trophoblast organoids [2,3] has significantly advanced research into early placenta development and related pregnancy complications. However, these model systems are derived from first-trimester placentas or blastocysts, whereas symptoms of placentation disorders, such as severe preeclampsia and fetal growth restriction, typically appear later in pregnancy [4,5]. Consequently, the health status of the original tissue used to derive these models is uncertain, and this may interfere with the study of developmental processes affected in these disorders.

Efforts have been made to generate TSCs from alternative sources, including naïve or primed pluripotent stem cells (PSCs), and adult somatic cells such as skin fibroblasts, term cytotrophoblasts, and umbilical-cord-derived mesenchymal stem cells (MSCs) [6,7,8,9,10,11,12,13,14,15,16,17,18,19,20,21,22,23,24] (recently reviewed in [25,26,27]). These somatic cells can be reprogrammed into induced PSCs (iPSCs), and subsequently converted into induced TSCs (iTSCs). Alternatively, reprogramming can be directed to iTSCs at an early stage, without fully transitioning through an iPSC state [9,10]. Recently, derivation of TSCs from term cytotrophoblasts without reprogramming has been reported [28]; however, due to the inherent susceptibility of term cytotrophoblasts to genetic mutations and chromosomal aberrations [29], the generation of iTSCs from alternative somatic cell types may offer a more advantageous and reliable approach. Besides the fact that these models overcome ethical limitations of using first-trimester placentas or blastocysts, they enable investigation of the pathogenesis of placentation disorders using disease-linked trophoblasts. This approach has recently been utilized by Morey et al., who compared iTSCs, generated via iPSCs, from umbilical cords of healthy and preeclamptic pregnancies [24].

Since the field is rapidly evolving, multiple approaches have been used to generate iTSCs, with different combinations of source cell types and methods for reprogramming and conversion into TSCs [25,26,27]. These combinations have had varying outcomes in terms of efficiency and TSC characteristics. In the current study, we optimized the generation of iTSCs from term umbilical-cord-derived MSCs, enabling non-invasive collection of patient-derived material immediately after birth. We used a direct reprogramming approach previously described for dermal fibroblasts by Tan et al. [21]. In this approach, cells are transduced with the OKSM reprogramming factors (OCT4, KLF4, SOX2, and MYC), and day-8 reprogramming intermediates are then directly transited into human TSC medium [1] to induce TSC conversion. Direct reprogramming is not only time-efficient, it also potentially minimalizes the erasure of epigenetic marks, which is a major challenge with cellular reprogramming [30]. During the first days of iTSC conversion, we added either A-485, bone morphogenetic protein 4 (BMP4), or EPZ-6438 (further referred to as EPZ) to the TSC medium to evaluate whether these compounds enhance TSC induction.

A-485 is a catalytic inhibitor of the acetyltransferases CREBBP/EP300 and has been shown to suppress pluripotency and reprogramming into iPSCs [31]. In addition, it blocks TSC differentiation into extravillous trophoblasts (EVTs) and syncytiotrophoblasts (STBs) [32]. Therefore, A-485 might promote the conversion of reprogramming intermediates into proliferating iTSCs. BMP4, a member of the TGF-β superfamily, plays a role in the emergence of trophectoderm and makes PSCs permissive to trophoblast conversion [33]. EPZ is an inhibitor of the enzymatic component of the polycomb repressive complex 2 (PRC2), which is an H3K27 methyltransferase that maintains naïve pluripotency and restricts differentiation into trophectoderm and mesoderm [34]. Some studies report that the short-term addition of BMP4 or inhibition of PRC2 in TSC medium enhances the efficiency of TSC conversion [15,16,20]. Therefore, these treatments might also boost the conversion into TSCs in our iTSC generation approach.

We assessed whether the generated iTSCs fulfilled the criteria for bona fide first-trimester trophoblasts as established by Lee et al. [35] and Karvas et al. [26]. These include protein expression of keratin 7 (KRT7), GATA binding protein 3 (GATA3), and transcription factor AP-2 gamma (TFAP2C); expression of miRNAs from the chromosome 19 miRNA cluster (C19MC); low levels of HLA type I surface molecules; hypomethylation of the E74-like ETS transcription factor 5 (*ELF5*) promoter; long-term self-renewal; and the ability to differentiate into invasive EVTs and hormone-producing STBs. Additionally, we evaluated the capacity of the iTSCs to form organoids with EVT differentiation potential. Finally, we compared the transcriptomes of the iTSCs with TSC lines that we previously derived from first-trimester placental tissue [32]. Our findings demonstrate that direct reprogramming of umbilical cord cells successfully generates bona fide iTSCs, while the tested supplements do not improve iTSC induction in this approach.

## 2. Results

### 2.1. Generation of iTSCs and Expression of Trophoblast Markers

**Figure 1:**
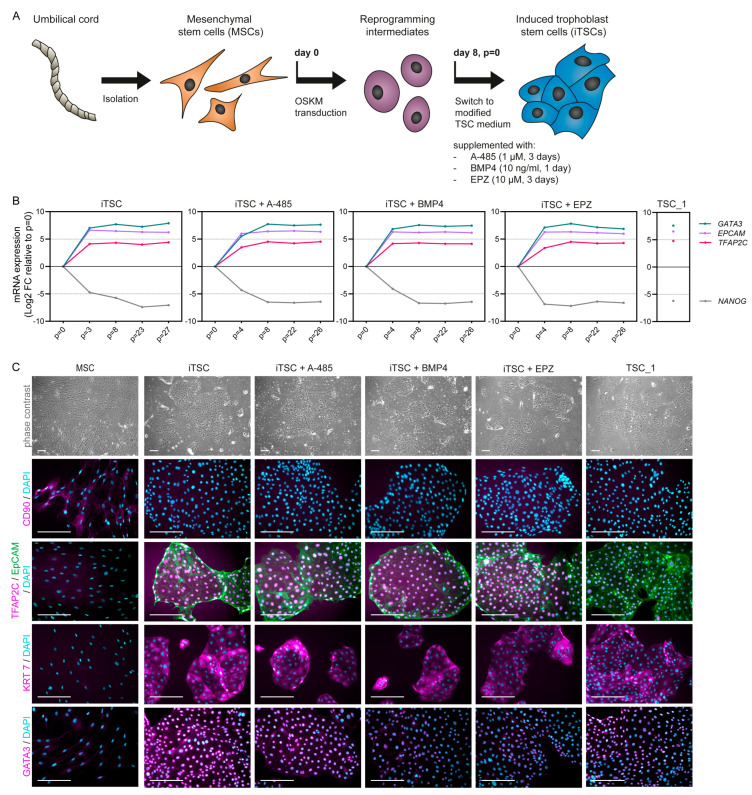
Induced trophoblast stem cells (iTSCs) generated from umbilical cord show trophoblast stem cell (TSC)-like marker expression and morphology: (**A**) Schematic representation of iTSC generation from umbilical cord using a direct reprogramming approach. During conversion of reprogramming intermediates (passage number (*p*) = 0) into iTSCs, the supplements A-485, BMP4, or EPZ were added for 1–3 days; (**B**) mRNA expression of *GATA3* and *TFAP2C* (trophoblast markers), *EPCAM* (epithelial cell marker), and *NANOG* (pluripotency marker) at different stages during conversion into iTSCs with and without the supplements, measured by qPCR and normalized to expression of housekeeping genes *GUSB* and *EEF2*. Expression levels in TSC_1, derived from first-trimester placental tissue, are shown for reference. Data points represent log2 fold-change (FC) relative to iTSC *p* = 0; (**C**) representative phase-contrast and immunofluorescence images of source umbilical cord mesenchymal stem cells (MSCs) (negative control for trophoblast markers), the iTSCs with different supplements (*p* = 33–35), and TSC_1 (positive control for trophoblast markers) stained for CD90 (fibroblast marker, magenta), EpCAM (epithelial cell marker, green), TFAP2C, KRT7, or GATA3 (trophoblast markers, magenta), with 4′,6-diamidino-2-phenylindole (DAPI) (nuclei, cyan). Scale bars: 200 µm.

MSCs were isolated from umbilical cord of an uncomplicated, term pregnancy and transduced with OKSM transcription factors on day 0 (Figure 1A). On day 8 (iTSC passage number (*p*) = 0), the reprogramming intermediates were transited into modified TSC medium [36] to induce iTSC conversion, with or without supplementation of A-485, BMP4, or EPZ for 1–3 days. The obtained cells could be cultured for at least 48 passages in TSC medium, indicating long-term self-renewal capacity. We measured mRNA expression of pluripotency and trophoblast markers at different time points during conversion into TSCs. At *p* = 0, expression of the pluripotency marker nanog homeobox (*NANOG*) could be detected but this gradually decreased with TSC induction (Figure 1B). In contrast, expression of the trophoblast markers *GATA3* and *TFAP2C* and the epithelial cell marker epithelial cell adhesion molecule (*EPCAM*) increased when cultured in TSC medium. Around *p* = 8, the markers reached similar mRNA levels as in TSCs derived from first-trimester placental tissue (TSC_1). In addition, the iTSCs obtained cobblestone-like morphology similar to that of TSC_1, and they expressed the trophoblast markers GATA3, KRT7, and TFAP2C on the protein level (Figure 1C), which is one of the criteria for first-trimester trophoblasts. As expected, the source MSCs were negative for these trophoblast markers and positive for the fibroblast marker CD90 (Figure 1C). There were no obvious differences in marker expression and cell morphology between the iTSC conditions (iTSC, iTSC + A-485, iTSC + BMP4, and iTSC + EPZ).

### 2.2. Expression of C19MC miRNAs, HLA Type I Profile, and Methylation Status of the ELF5 Promoter

**Figure 2:**
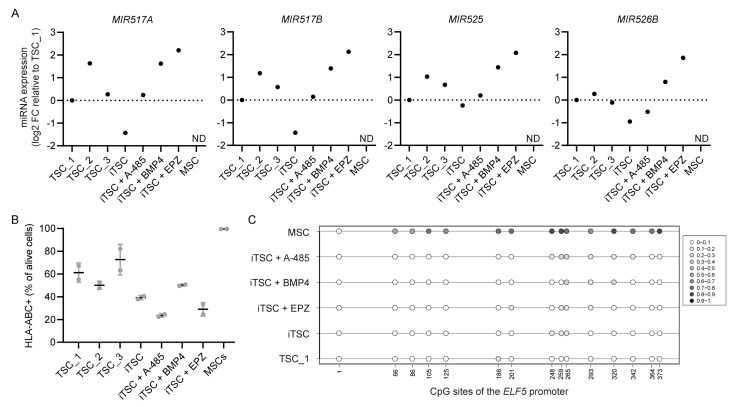
All iTSC conditions generated cells that express miRNAs from the chromosome 19 miRNA cluster (C19MC), have decreased HLA-ABC expression, and show hypomethylation of the *ELF5* promoter: (**A**) Expression of four C19MC miRNAs in iTSCs (*p* = 25–27), measured by qPCR and normalized to expression of *MIR103A*. Data points represent log2 fold-change (FC) relative to TSC_1. In MSCs, expression was not detectable (ND); (**B**) number of HLA-ABC-positive cells as percentage of total number of alive cells, measured by flow cytometry. The mean is shown of two independent experiments using different passage numbers (iTSCs *p* = 40–45); (**C**) methylation status of CpG sites at the *ELF5* promoter, determined by bisulfite sequencing (n = 8 clones per condition, iTSCs *p* = 8–9). Closed circles: methylated; open circles: non-methylated. The color shade displays the ratio of clones methylated at that CpG site.

After the generation of iTSCs, we examined them for additional trophoblast criteria. Expression of the C19MC genes *MIR517A, MIR517B, MIR525*, and *MIR526B* was not detectable in the source MSCs, whereas these genes were expressed in all iTSC conditions (Figure 2A). The expression levels somewhat differed between iTSC conditions but similar variability was present among TSC lines derived from first-trimester placenta (TSC_1-3). The miRNA expression levels were highest in the iTSC condition supplemented with EPZ and lowest in the iTSC condition without supplement. Most importantly, compared with the MSCs, expression of C19MC miRNAs had clearly increased during iTSC conversion under all iTSC conditions.

Regarding the HLA type I profile, we found that almost 100% of the MSCs were positive for HLA-ABC in flow-cytometric analysis, while this percentage decreased to ~20–50% in the iTSCs (Figure 2B, see Figure S1 for gating strategy). Interestingly, this percentage was lower than the percentage of HLA-ABC-positive cells in TSC_1-3, which was relatively high (~50–80%). The percentage of HLA-ABC-positive cells was lowest in the iTSC conditions supplemented with A-485 or EPZ.

Bisulfite sequencing showed that all iTSC conditions induced hypomethylation of the *ELF5* promoter as compared with the source MSCs (Figure 2C). Among the iTSC conditions, the condition without supplement had the lowest methylation levels and was closest to TSCs derived from first-trimester placenta, although differences were very minor. Overall, all iTSC conditions generated cells that met the criteria for first-trimester trophoblasts proposed by Lee et al. [35].

### 2.3. Differentiation and Organoid Formation Capacity

**Figure 3:**
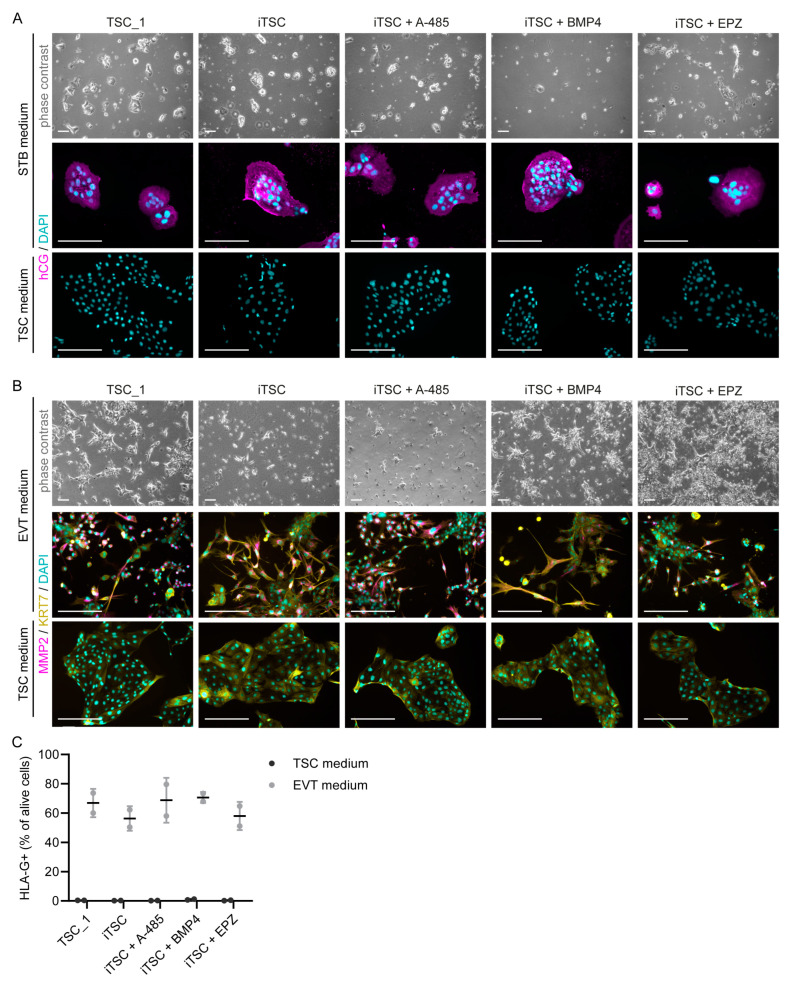
All iTSC conditions generated cells that can undergo syncytiotrophoblast (STB) and extravillous trophoblast (EVT) differentiation. Representative phase-contrast and immunofluorescence images of (**A**) iTSCs in STB medium for 4 days, stained for hCG (STB marker, magenta) with DAPI (nuclei, cyan), and (**B**) iTSCs in EVT medium for 5 days, stained for MMP2 (EVT marker, magenta), KRT7 (trophoblast marker, yellow), and DAPI (nuclei, cyan). The corresponding cells in TSC medium are also shown with the same stainings (negative controls for STB/EVT marker). iTSCs *p* = 33–35. Scale bars: 200 µm. (**C**) Percentage of HLA-G-positive cells in TSC and EVT medium measured by flow cytometry. The mean is shown of two independent experiments using different passage numbers (iTSCs *p* = 40–45).

To further investigate functional characteristics of the iTSCs, we induced monolayer STB and EVT differentiation and organoid formation. Although the morphology of differentiated cells was somewhat variable between iTSC conditions, from all iTSC conditions we were able to generate multinucleated STBs expressing the STB marker human chorionic gonadotropin (hCG) (Figure 3A), and EVTs expressing the EVT markers matrix metalloproteinase-2 (MMP2) and major histocompatibility complex, class I, G (HLA-G) (Figure 3B,C), as shown by immunostainings and flow cytometry.

**Figure 4:**
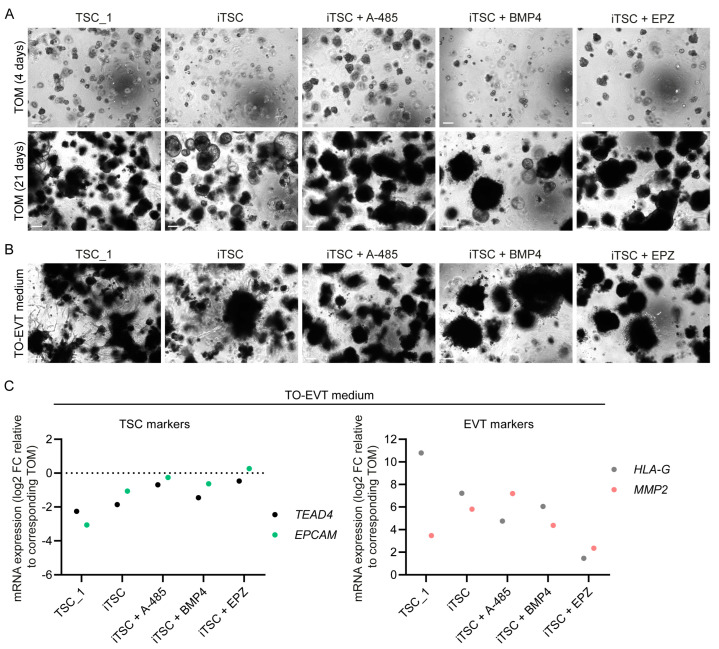
Not all iTSC conditions generated cells that can efficiently form organoids and EVT outgrowth. Representative phase-contrast images of organoids (**A**) after 4 days and after 21 days in trophoblast organoid medium (TOM) and (**B**) after 4 days in TOM followed by 17 days in trophoblast organoid (TO)–EVT medium. Organoid formation and differentiation were performed in two independent experiments with different passage numbers of the cells, with similar morphological results (*p* = 32–38). Scale bars: 200 µm. (**C**) mRNA expression of TSC and EVT markers, measured by qPCR, after 4 days in TOM followed by 17 days in TO–EVT medium (as shown in panel (**B**)). Data points are normalized to expression of *GUSB* and *EEF2* and represent log2 fold-change (FC) relative to the corresponding condition in TOM for 21 days.

In addition, iTSCs from all conditions could form organoids when cultured in a Matrigel matrix in trophoblast organoid medium (TOM), indicating stemness (Figure 4A). Again, the morphology was somewhat diverse between conditions, and the BMP4 condition only formed a few, very large organoids. When cultured in trophoblast organoid (TO)–EVT medium, the iTSC condition without supplement formed outgrowth of invasive EVTs (Figure 4B). However, in the A-485, BMP4, and EPZ conditions, the EVT outgrowth seemed to be reduced, which may indicate reduced EVT differentiation and/or invasion capacity. For the EPZ condition in particular, reduced EVT differentiation was reflected in TSC and EVT marker expression, which was almost unchanged compared to the corresponding organoids in TOM (Figure 4C). The other conditions all upregulated EVT markers, suggesting that the observed reduction in EVT outgrowth was a result of limited invasion capacity rather than impaired EVT differentiation.

### 2.4. Transcriptome Analysis

**Figure 5:**
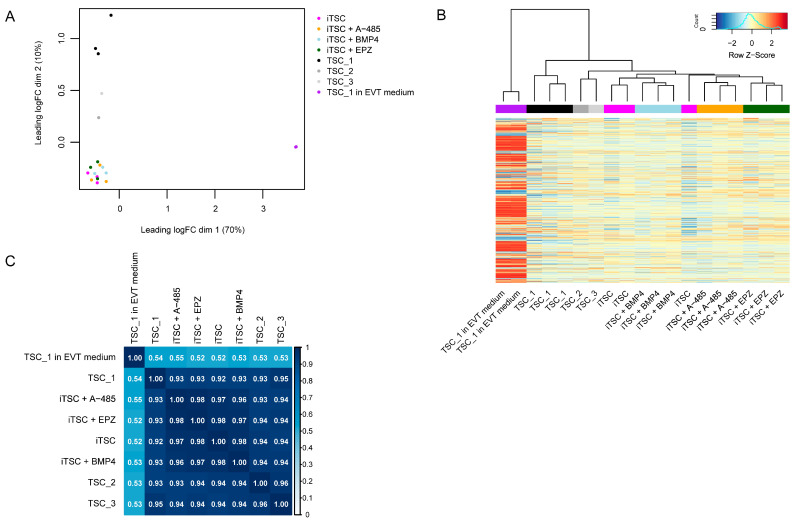
The iTSC conditions and TSCs derived from first-trimester placenta are similar in trophoblast gene signature: (**A**) hierarchical clustering across samples; (**B**) multidimensional scaling plot showing sample clustering; (**C**) correlation plot using R-squared values of group means. All analyses were based on log2 counts per million of trophoblast- and/or placenta-enriched genes. TSC_1-3 are derived from first-trimester placenta. For TSC_1 (n = 3), TSC_1 in EVT medium (n = 2, 4 days in EVT medium), and the iTSC conditions (n = 3, *p* = 34–47), replicates are different passage numbers of the same lines.

Lastly, we performed transcriptome analysis to examine the similarity between the iTSCs and TSCs derived from first-trimester placental tissue, and across iTSC conditions. EVTs generated from TSC_1 were also included for reference. As we were predominantly interested in the trophoblast signature, we specifically looked at trophoblast- and placenta-enriched genes. For this, we used a list of genes that are elevated (by at least four-fold in mRNA level) in trophoblast and/or placenta compared to other cell types and tissues, respectively, downloaded from the Human Protein Atlas [37,38]. As expected, EVTs generated from TSC_1 clustered separately from all other groups (Figure 5A,B). TSC_1-3 also separated slightly from the iTSCs (Figure 5A,B) but correlations were still high (Figure 5C), indicating that the iTSCs were similar to first-trimester placenta-derived TSCs based on their transcriptomes. Across the iTSC conditions, there was high overlap (Figure 5A,B) and high correlation (Figure 5C), indicating no obvious effects of the supplements.

## 3. Discussion

In this study, we optimized the generation of human iTSCs from term umbilical-cord-derived MSCs and examined the generated iTSCs for criteria of bona fide first-trimester trophoblasts. We used a direct reprogramming approach previously established for dermal fibroblasts, in which we investigated the effect of three supplements—A-485 (CREBBP/EP300 acetyltransferase inhibitor), BMP4 (growth factor), and EPZ (PRC2 methyltransferase inhibitor)—to assess their potential to enhance iTSC induction. The generated iTSCs exhibited key trophoblast characteristics and functional capacities, comparable to TSCs derived from first-trimester placental tissue, and thus provide a robust model for studying early placental development and the pathogenesis of placentation disorders using patient-derived trophoblasts. Our results showed no evidence that any of the supplements boosted iTSC induction.

Successful transition from reprogramming intermediates into TSCs was confirmed by the upregulation of trophoblast markers and downregulation of pluripotency markers. The iTSCs could be cultured long-term, and bona fide first-trimester trophoblast identity was further demonstrated by the appearance of trophoblast marker expression on protein levels, C19MC miRNA expression, downregulation of HLA type I surface molecules, and hypomethylation of the *ELF5* promoter as compared to the source MSCs. Although each iTSC condition met these criteria, minor differences in marker levels across conditions suggest that the supplements introduced subtle variations that persisted through several passages after removal of the supplements; however, these differences are unlikely to significantly compromise the utility of the iTSCs for modeling early placental development and disease phenotypes.

Although morphology was somewhat variable, the differentiation capacity into STBs and EVTs was robust across all iTSC conditions. The ability of these cells to proliferate long-term under stem cell conditions and differentiate into STBs and EVTs under differentiation-inducing conditions is an advantage of the use of TSC medium over BMP4-directed trophoblast induction, which results in mixed trophoblast populations with limited self-renewal capacity [23,39,40,41,42]. Additionally, all iTSC conditions were able to form trophoblast organoids. The formation of trophoblast organoids from iTSCs derived from naïve PSCs or dermal fibroblasts has previously been demonstrated [10,12,18]. However, to the best of our knowledge, this has not yet been achieved using umbilical cord MSC-derived iTSCs or through direct reprogramming. Organoids of the supplemented conditions, particularly the EPZ condition, exhibited reduced EVT differentiation and/or invasion capacity. Since trophoblast differentiation into invasive EVTs plays a critical role in placentation disorders, such as preeclampsia and fetal growth restriction [4,5], the unsupplemented iTSC condition offers a more suitable model for investigating the pathogenesis of these diseases, allowing for the study of EVT invasion.

Transcriptome analysis revealed a high correlation in the expression of trophoblast- and placenta-enriched genes across the iTSC conditions, indicating that the overall trophoblast transcriptional signature was not affected by the supplements. In addition, transcriptomes of the iTSCs displayed strong resemblance to those of TSCs derived from first-trimester placental tissue, underscoring the effectiveness of this protocol in obtaining TSCs. We have previously shown that A-485 keeps TSCs in a proliferative stem cell state [32], and A-485 prevents reprogramming of iPSCs [31]. Even though both phenomena could have led to more efficient conversion into iTSCs in the presence of A-485, we did not observe this. Other studies have reported enhanced TSC induction from PSCs by short-term BMP4 addition [15,16] or PRC2 inhibition/functional knock-out [15,20] based on the higher expression of trophoblast markers, a stronger decrease in pluripotency marker expression, or less morphological heterogeneity within the cultures. However, in our iTSC generation approach, BMP4 and EPZ had limited impact on TSC induction. This discrepancy may stem from differences in the starting cell population, as the aforementioned protocols used naïve or primed PSCs, whereas we used reprogramming intermediates. The reprogramming intermediates may already efficiently convert into TSCs without the need for these supplements.

One limitation of our study is the use of a single donor umbilical cord for the generation of iTSCs, which precluded statistical testing and may limit the generalizability of our findings. Repeating this protocol with a larger sample size is essential to validate reproducibility and determine whether individual differences between donors affect the efficiency of iTSC generation.

Additionally, we did not evaluate whether the iTSCs retained donor-specific epigenetic marks, which is crucial for accurately modeling patient-specific disease phenotypes. Naïve PSC-derived iTSCs may lose donor-specific methylation patterns due to global methylation loss during naïve conversion [43,44]. However, Cinkornpumin et al. and Karvas et al. showed that these DNA methylation patterns are largely regained during the conversion into iTSCs, although some aberrant DNA hypomethylation persisted [8,18]. Primed-state iPSCs, in contrast, tend to retain donor-specific DNA methylation patterns [27]. Interestingly, Morey et al. showed that iTSCs derived from preeclampsia-associated primed iPSCs—but not isogenic naïve ones—exhibited a preeclampsia phenotype, suggesting that disease-associated, donor-specific marks are retained in primed but not naïve iPSC-derived iTSCs [24]. Conventional OKSM reprogramming generates primed-state iPSCs, which resemble post-implantation epiblast cells [45,46]. Buckberry et al. showed that the most significant changes in DNA methylation take place between days 13 and 21 after OKSM transduction [47]. The direct reprogramming approach that we utilized may minimalize the erasure of patient-specific epigenetic marks as cells do not go beyond day 8 of reprogramming nor through a naïve state [27]; however, further investigation of this is required.

In conclusion, we successfully generated bona fide iTSCs from term umbilical cord using a direct reprogramming approach, providing a model to study early placentation mechanisms in patient-specific trophoblasts. The use of umbilical-cord-derived MSCs enables the collection of patient-derived material immediately after birth, without the need for obtaining a skin biopsy. As an additional benefit, iTSCs overcome the ethical limitations of using first-trimester placental tissue or blastocysts as the source of TSCs. Moreover, the possibility to generate iTSCs and iPSCs from the same donor provides a powerful tool for investigating trophoblast interactions with other placental cell types, such as endothelial and immune cells, and embryonic lineages. Overall, this study represents an advancement in trophoblast research by providing a clinically relevant in vitro model for understanding the complexities of early placental development and mechanisms of placental disease.

## 4. Materials and Methods

### 4.1. Tissue Collection and MSC Culture

Collection of umbilical cord and generation of iPSCs were approved by the Amsterdam UMC Biobank review committee (2023.07/2021.0449). Umbilical cord tissue was collected from an uncomplicated, term pregnancy with written informed consent of the mother. MSCs were obtained from the umbilical cord and cultured similarly as described by Horii et al. [42]. In short, the umbilical cord tissue was washed in PBS containing 2% penicillin/streptomycin (Gibco, Waltham, MA, USA, 15140122) and 0.5% gentamicin (Gibco, 15710049), and cut into 1–2 mm fragments, which were plated at regular intervals on tissue culture dishes. After adhering for 30–60 min at room temperature, the fragments were overlayed with DMEM high glucose (Gibco, 41966029) with 10% FBS (Gibco, 10270106), 1% penicillin/streptomycin, and 0.5% gentamicin, which was refreshed every 3–4 days. The plates were kept at 37 °C in a 5% CO_2_ humidified atmosphere. The fragments were regularly checked for fibroblast outgrowth. When confluent outgrowth was observed around multiple fragments, the fibroblasts were passaged using 0.25% Trypsin and expanded in fibroblast medium (DMEM high glucose, GlutaMAX supplement, pyruvate (Gibco, 31966021) with 10% FBS, 1X MEM Non-Essential Amino Acid Solution (Gibco, 11140035), 1% penicillin/streptomycin, and 55 µM 2-mercaptoethanol (Gibco, 31350010)). The MSCs were stored in Cell Banker 1 (AMSBIO, Cambridge, MA, USA, 11910) in liquid nitrogen until further processing.

### 4.2. Generation of iTSCs

Following the protocol of Tan et al. [21], MSCs were directly reprogrammed into iTSCs. Briefly, the cells were seeded on day -2 in fibroblast medium, and transduced on day 0 with integration-free Sendai viruses containing the reprogramming factors KOS (KLF4, OCT4, SOX2), c-MYC and KLF4 (respective MOIs of 5:5:6) from the CytoTune-iPSC 2.0 Sendai reprogramming kit (Invitrogen, Waltham, MA, USA, A16517) for 24 h. The plates were kept at 37 °C in a humidified atmosphere with 5% O_2_ and 5% CO_2_, and the fibroblast medium was refreshed every other day. On day 7, the reprogramming intermediates were passaged using TrypLE onto irradiated mouse embryonic fibroblasts (iMEFs) in fibroblast medium. At day 8, the cells were transited into modified TSC medium (DMEM/F12 + Glutamax supplemented with 1% KSR, 0.5% penicillin/streptomycin, 0.15% BSA, 1% ITS-X, 200 µM L-ascorbic acid, 50 ng/mL human recombinant EGF, 2 µM CHIR99021, 5 µM A83-01, 0.8 mM VPA, and 2.5 µM Y-27632) [36]. At this point, the TSC medium was supplemented with either 1 µM A-485 (MedChemExpress, Monmouth Junction, NJ, USA, HY-107455) for 3 days, 10 ng/mL BMP4 (BioLegend, San Diego, CA, USA, 795606) for 1 day, or 10 µM EPZ-6438 (SelleckChem, Houston, TX, USA, S7128) for 3 days. The TSC medium was refreshed every day until cobblestone-shaped colonies emerged. Cells were then passaged every 4–5 days and refreshed every 2–3 days. The iTSCs were cultured on iMEFs for 3 passages before changing to 5 µg/mL mouse-collagen-IV-coated plates.

### 4.3. TSC/Organoid Culture and Differentiation

TSC culture, monolayer EVT and STB differentiation, trophoblast organoid formation, and organoid–EVT differentiation were performed as described previously [32], except that the culture of TSCs and monolayer differentiation were performed under low oxygen conditions (5% O_2_).

### 4.4. Standard Analyses

Total RNA isolation, quantitative real-time PCR (qPCR), immunofluorescent staining, and microscopy were performed as described previously [32]. See Table S1 for the primer sequences and Table S2 for antibodies used.

### 4.5. qPCR of miRNAs

qPCR of miRNAs was performed as described by others [35]. See Table S1 for the primer sequences.

### 4.6. Flow Cytometry

Cells were dissociated using TrypLE for 20 min at 37 °C. Cell suspensions were stained using FITC-conjugated anti-HLA-G and APC-conjugated anti-HLA-ABC antibodies (see Table S2) for 15 min at room temperature, and Bioscience Fixable Viability Dye eFluor 780 (Invitrogen, 65-0865-14) for another 15 min at room temperature. Unstained cells were used as control. Flow cytometry was performed using an SP6800 Spectral Cell Analyser (Sony, Tokyo, Japan) and data were analyzed using FlowJo software (v10). Doublets and death cells were excluded from analysis.

### 4.7. Bisulfite Sequencing

Methylation analysis of the *ELF5* promoter by bisulfite conversion and Sanger sequencing were performed as described by Lee et al. [17]. Methylation data were visualized using the web tool Methylation Plotter [48].

### 4.8. Transcriptome Sequencing

Total RNA libraries were prepared using the mRNA Capture KAPA HyperPrep kit (Roche, Basel, Switzerland, 0000162206), after which 150 bp paired-end sequencing was performed on an Illumina NovaSeqXPlus system. Initial quality control was conducted using FastQC (v0.11.8) and FastQ Screen (v0.14.1). Reads were quality-trimmed, and adapters were removed using Trim Galore (v0.6.6). The processed reads were analyzed to obtain transcript-level abundance estimates in transcripts-per-million using Salmon (v1.9.0) in quasi-mapping-based mode, with the GRCh38 transcriptome (release 107) as the reference. All downstream analyses were performed using the R programming language (v4.2.2). Following quantification, transcript-level abundance values were summarized to gene-level counts using the tximport package (v1.32.0), and gene-level analyses were performed with the Bioconductor package EdgeR (v4.2.1).

### 4.9. Data Availability

Transcriptome sequencing data were deposited in the Gene Expression Omnibus database under accession number GSE282356.

## Data Availability

The original transcriptome sequencing data presented in the study are openly available in the Gene Expression Omnibus at GSE282356.

## Supplementary Materials

The supporting information can be downloaded at: https://www.mdpi.com/article/10.3390/ijms26010271/s1.
