# Supplementary material for: Generation of Bona Fide Human Induced Trophoblast Stem Cells by Direct Reprogramming of Term Umbilical Cord Cells
Source: Int J Mol Sci. 2024 Dec 31;26(1):271. doi: 10.3390/ijms26010271 (PMC11719488; doi:10.3390/ijms26010271)
Supplement: Supplementary file 1 [file ijms-26-00271-s001.zip › ijms-3369619-supplementary.pdf]

Supporting Information for

Generation of Bona Fide Human Induced Trophoblast Stem Cells by Direct Reprogramming of Term Umbilical Cord Cells

A. Jantine van Voorden [1,3], Souad Boussata [1,3], Remco Keijser [1,3], Marloes Vermij [1,3], Muriel K. Wagner [1,3], Wessel Ganzevoort [2,3], Gijs B. Afink [1,3,4,*]

1 Reproductive Biology Laboratory, Amsterdam University Medical Center location University of Amsterdam, Meibergdreef 9, 1105 AZ, Amsterdam, the Netherlands.
2 Department of Obstetrics and Gynaecology, Amsterdam University Medical Center location University of Amsterdam, Meibergdreef 9, 1105 AZ, Amsterdam, the Netherlands.
3 Amsterdam Reproduction and Development Research Institute, Amsterdam, the Netherlands.
4 Amsterdam Institute for Immunology and Infectious Diseases, Amsterdam, the Netherlands.
* Correspondence: g.b.afink@amsterdamumc.nl

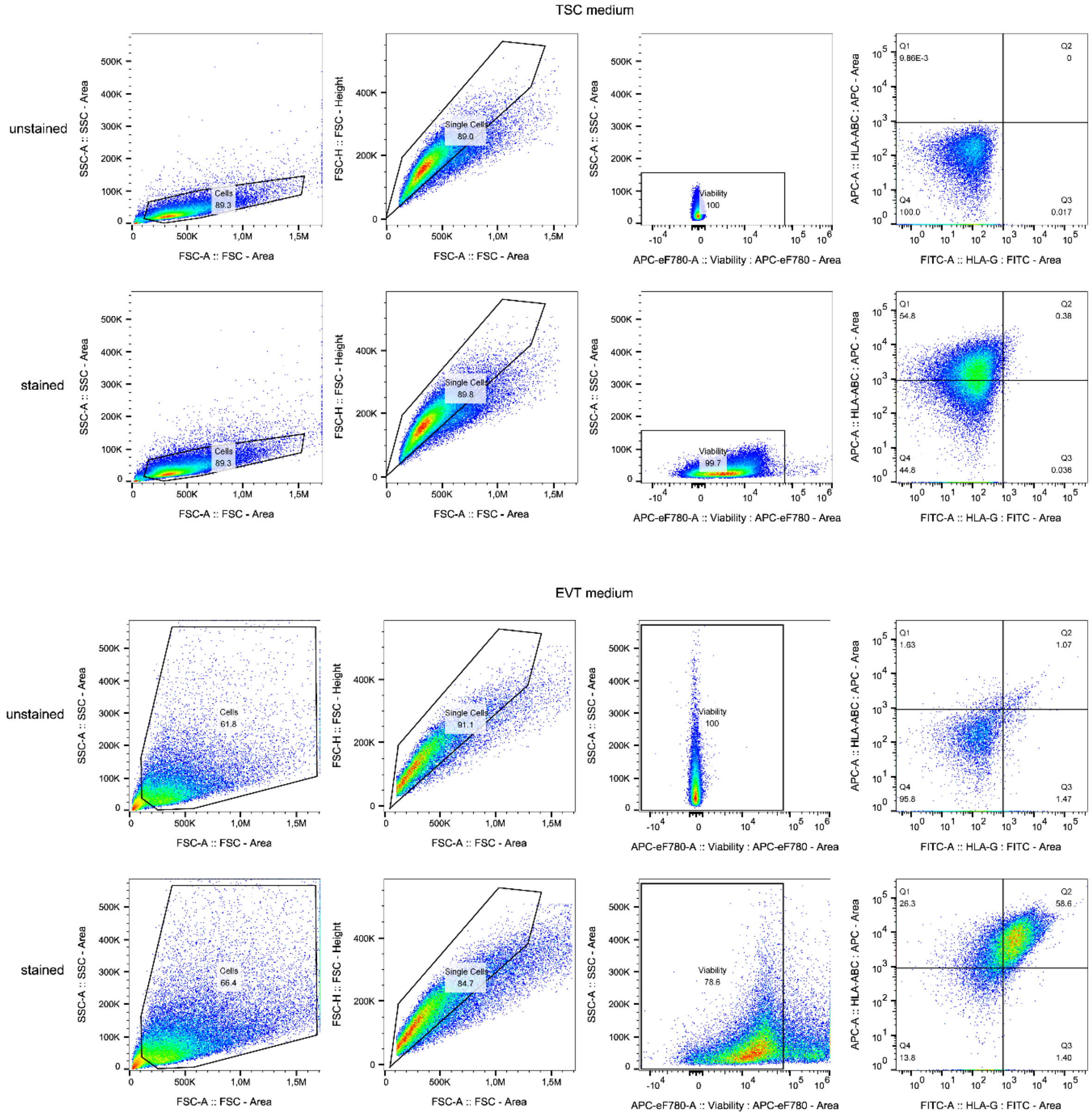

Figure S1. Gating strategy used for HLA-ABC and HLA-G FACS. Representative images of unstained and stained cells in TSC medium and EVT medium are shown.

Table S1. Primer sets used for quantitative real-time PCR.

Target gene symbol	Forward primer	Reverse primer
EEF2	CTGGAGATCTGCCTGAAGGA	CGACCGGGTCAGATTTCTT
EPCAM	GCCAGTGTACTTCAGTTGGTGC	CCCTTCAGGTTTTGCTCTTCTCC
GATA3	CTACTACGGAAACTCGGTCAGGGC	AGCCAGGGTAGGGATCCATGAAG
GUSB	GGAGTGCAAGGAGCTGGAC	ATTGAAGCTGGAGGGAACTG
HLA-G	CCACCACCCTGTCTTTGACTAT	ACGTCCTGGGTCTGGTCCT
MIR103A	GTAGCAGCATTGTACAGGG	GTTGGCTCTGGTGCAGGGTCCGAGGTATTCGCACCAG AGCCAACTCATAG
MIR517A	GTTTGGATCGTGCATCCTTTTA	GTTGGCTCTGGTGCAGGGTCCGAGGTATTCGCACCAG AGCCAACACACTC
MIR517B	GTGCCTCTAGATGGAAGCA	GTTGGCTCTGGTGCAGGGTCCGAGGTATTCGCACCAG AGCCAACAGACAG
MIR525	GTTGAAGGCGCTTCCCTTT	GTTGGCTCTGGTGCAGGGTCCGAGGTATTCGCACCAG AGCCAACCGCTCT
MIR526B	GTTTGGGAAAGTGCTTCCTTTT	GTTGGCTCTGGTGCAGGGTCCGAGGTATTCGCACCAG AGCCAACGCCTCT
MMP2	TGGCACCCATTTACACCTACAC	ATGTCAGGAGAGGCCCCATAGA
NANOG	TTTGTGGGCCTGAAGAAAACT	AGGGCTGTCCTGAATAAGCAG
TEAD4	CAGGTGGTGGAGAAAGTTGAGA	GTGCTTGAGCTTGTGGATGAAG
TFAP2C	CACCTGTTGCTGCACGATCAGA	AGGAGCGACAATCTTCCAGGGA

Table S2. Antibodies used for immunofluorescence (IF) and flow cytometry (FC).

Target protein	Manufacturer	Cat. no.	Source	Clone	Dilution	Application
CD90-FITC	Invitrogen	11-0909-42	mouse	5E10	1:50	IF
EpCAM	Cell Signaling Technology	2929	mouse	VU1D9	1:400	IF
EpCAM	Cell Signaling Technology	36746	rabbit	D4K8R	1:200	IF
GATA3	Invitrogen	MA-1028	mouse	1A12-1D9	1:200	IF
hCG	DAKO Omnis	A023102	rabbit	*polyclonal*	1:400	IF
HLA-G	ExBio	11-291-C100	mouse	MEM-G/1	1:100	IF
HLA-G-FITC	ExBio	1F-292-C100	mouse	MEM-G/9	1:200	FC
HLA-ABC-APC	eBioscience	17-9983-42	mouse	W6/32	1:20	FC
KRT7	Agilent Technologies	M701801-2	mouse	OV-TL 12/30	1:200	IF
MMP2	Cell Signaling Technology	40994S	rabbit	D4M2N	1:200	IF
TFAP2C	Santa Cruz	sc-12762	mouse	6E4/4	1:100	IF
